# P-475. Anal Cancer Screening in HIV Positive MSM and TGW: A Multistage Quality Improvement Project

**DOI:** 10.1093/ofid/ofae631.674

**Published:** 2025-01-29

**Authors:** David Kenison, Rudline G Zamor, Khushbu Shah, Sarath Nath

**Affiliations:** Stony Brook University Hospital, Lake Grove, New York; Stony Brook University Hospital, Lake Grove, New York; Stony Brook University Hospital, Lake Grove, New York; Stony Brook University Hospital, Lake Grove, New York

## Abstract

**Background:**

Men who have sex with men (MSM) and transgender women (TGW) living with HIV (PLHIV) have an estimated 37-fold increased risk of anal cancer compared to the general population. Currently there are no national guidelines on screening for anal cancer with cytological analysis creating discrepancies in anal cancer screening and increasing risk of progression to anal cancer. We reviewed the MSM and TGW PLHIV cohort in our institution to identify demographics, risk factors, and anal cancer screening rates, we also identified outcomes of patients with cytological testing.

**Figure:**
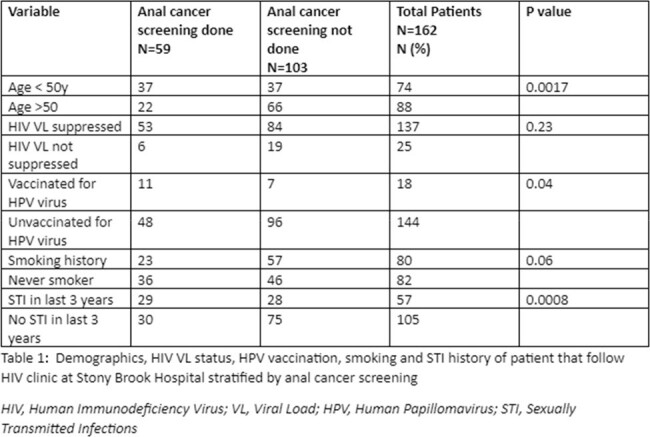

**Methods:**

We conducted a retrospective chart review of MSM PLHIV over the age of 18, who were seen in Stony Brook University HIV clinic from 02/01/21 to 02/01/24. Demographics, smoking history, HIV viral load, human papillomavirus (HPV) vaccination status, and sexually transmitted infection (STI) history was obtained from chart review and compared between patients with and without anal cancer screening with Chi square test.

**Figure:**
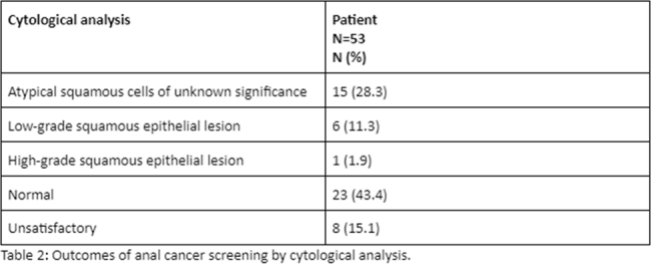

**Results:**

We identified a total of 160 MSM and 2 TGW. The majority of patients were unvaccinated for HPV (n=144; 88%). 84% had suppressed HIV viral load (n=137), 49% had a smoking history (n=80), and 35% had recent history of STI (n=57). Only 32% (n=53) patients had anal cancer screening done. We also found the patients who were less than 50 years, who had smoking history and recent STIs had a higher chance of being screened. Fifty-three patients were identified with prior cytological analysis, 15 (28.6%) with atypical squamous cells of unknown significance, 6 (11.3%) with low-grade, and 1 (1.9%) with high-grade squamous epithelial lesions.

**Conclusion:**

We identified that anal cancer screening rates were low at our institution despite the prevalence of risk factors such as smoking, high prevalence of STIs and low HPV vaccination status. Nearly 50% of patients had abnormal cytological analysis, highlighting the importance of establishing and implementing anal cancer screening. We have implemented an institutional protocol from 03/01/24 to increase the screening rates for anal cancer for this high risk population.

**Disclosures:**

**All Authors**: No reported disclosures

